# Immunomodulatory roles of regulatory T cells in cutaneous wound healing: mechanisms and therapeutic opportunities

**DOI:** 10.3389/fimmu.2026.1737438

**Published:** 2026-03-17

**Authors:** Samuel Emeka Peter, Farooq Riaz, Yikui Li, Xiaoli Zhao, Fan Pan

**Affiliations:** 1 Shenzhen Institutes of Advanced Technology (SIAT), Chinese Academy of Sciences (CAS), Shenzhen, China; 2 University of Chinese Academy of Sciences, Beijing, China; 3 Faculty of Pharmaceutical Sciences, Shenzhen University of Advanced Technology (SUAT), Shenzhen, China

**Keywords:** acute wound, chronic wound, cutaneous wound healing, inflammation, regulatory T cells, tissue repair

## Abstract

Cutaneous wound healing is a complex, tightly regulated biological process encompassing four overlapping phases: hemostasis, inflammation, proliferation, and remodeling. While acute wounds typically progress through these stages in a coordinated manner, various pathological conditions, including diabetes mellitus and microbial infections, can impair this process, resulting in chronic, non-healing wounds. A sustained inflammatory phase characterizes chronic wounds and is commonly associated with systemic immune dysregulation. Emerging evidence show that regulatory T cells (Tregs) are critical modulators of tissue homeostasis and regeneration. Tregs exert their effects through the expression of immunoregulatory molecules and the secretion of anti-inflammatory cytokines, facilitating the resolution of inflammation, supporting angiogenesis, and promoting tissue repair. In the context of cutaneous wounds, skin-resident Tregs interact with both immune and non-immune cells, contributing to the restoration of barrier integrity. This review highlights the multifaceted roles of Tregs in cutaneous wound healing, with a particular emphasis on their contributions to the inflammatory and proliferative phases, including vascularization and regulation of fibroblasts. Furthermore, emerging therapeutic strategies targeting Tregs to modulate their function in chronic wound settings are discussed. These insights underscore the potential of Tregs as novel immunotherapeutic targets for enhancing wound repair and regeneration in chronic and diabetic wound pathologies.

## 1 Introduction

The skin is the largest organ of the body, serving as a barrier and shield to protect the body from external environments, injuries, pathogens, and chemicals [1]. It performs physical protection, moisture retention, temperature regulation, external stimuli detection, and immunological modulation (1). Mammalian skin comprises two layers: the epidermis and the dermis, situated above the hypodermis and deeper fascia. The epidermis mainly consists of keratinocytes, along with the openings of hair follicles and sweat and sebaceous glands. These appendages penetrate the dermis, where diverse fibroblasts deposit collagen matrix and are essential for wound healing (1).

Cutaneous wounds are injuries to the skin and underlying tissue, primarily caused by conditions affecting the skin and subcutaneous tissue, such as diabetic foot ulcers, pressure ulcers, and venous leg ulcers. They often fail to heal within a typical duration, persisting for weeks or months (2). These wounds are especially prevalent in elderly individuals and those with chronic conditions such as diabetes or vascular disease (2). While mostly viewed as a clinical issue, chronic wounds have significant economic implications due to their high treatment costs and the lasting disability they cause (3). For economists, chronic wounds represent a substantial and increasing source of productivity reduction, healthcare expenditures, and social welfare strain globally (3–5).

The pathogenesis and outcomes of cutaneous wounds determine their categorization into acute and chronic wounds (6). A typical acute wound progresses through a well-defined sequence of stages: hemostasis involves the initial prevention of blood loss by wound clotting, the inflammation phase, which consists of the clearance of pathogens in the wound bed, formation of granulation tissue and re-epithelialization, termed the proliferation stage, and ultimately, the remodeling stage that comprises of collagen remodeling accompanied by vascular maturation and regression. Depending on the degree of injury, acute wounds can last from a few days to several months (7, 8). On the contrary, chronic wounds constitute a defective system that does not follow a coordinated manner. Chronic wounds often fail to progress past the inflammatory stage, characterized by prolonged inflammation, elevated reactive oxygen species (ROS), immune cell dysfunction, exudation, repeated infection, tissue necrosis, impaired keratinocyte migration, defective re-epithelialization, and decreased angiogenesis (9–12). Individuals suffer from diverse chronic wound types such as diabetic foot ulcers, pressure ulcers, and arterial ulcers, making the condition a significant global concern. Nearly 2.5 percent of Americans suffer from chronic wounds, which significantly impair their quality of life and place a heavy strain on society (13, 14).

Cutaneous wound healing is a complex yet sequentially overlapping process that occurs through four distinct stages: hemostasis, inflammation, proliferation, and dermal remodeling, ultimately resulting in the physiological and architectural restoration of the skin following damage (15). This intricate multicellular biological process involves a coordinated response from innate and adaptive immune cells, platelets, fibroblasts, epithelial cells, and endothelial cells (1, 16, 17). These immune cells restore the integrity of the skin by secreting various molecules that signal to local tissue progenitors and stromal cells, promoting wound repair.

Both immune cells (neutrophils, macrophages, mast cells, and B and T lymphocytes) and non-immune cells (endothelial cells and fibroblasts) are collectively involved in tissue inflammation and regeneration (18–20). Emerging evidence indicates that Tregs, a CD4+ T cell subset, play a central role in tissue repair by regulating immune responses at the site of injury through the secretion of various growth factors and cytokines necessary for wound healing (21, 22). In recent years, Treg-centered therapeutic approaches have held significant potential in advancing regenerative medicine, particularly by enhancing cutaneous wound healing and tissue restoration through their immunosuppressive functions and ability to attenuate inflammatory responses (10, 23–25).

In this review, we explore the emerging role of Tregs in cutaneous wound healing, focusing on their immunological mechanisms and contributions to tissue repair. We describe the mechanism by which Tregs orchestrate immune regulation at the site of injury. We further discuss the intrinsic and extrinsic factors that influence Treg differentiation, activation, and recruitment to wounded tissue, including chemokine signaling, metabolic conditions, and the local cytokine milieu. Finally, we highlight current and potential strategies aimed at enhancing Treg accumulation and function in the wound microenvironment, such as adoptive Treg transfer, cytokine-based therapies, and biomaterial-mediated delivery systems, positioning Treg-targeted approaches as a promising therapy in regenerative medicine and chronic wound management.

## 2 Immune mechanisms in healthy skin and wound healing

The skin plays a crucial role in maintaining homeostasis and acting as a barrier against external stimuli. Preserving skin integrity is vital for overall health, as injuries from chronic conditions, burns, trauma, or surgical procedures can lead to physical impairment (26). The skin’s structure is stratified into three layers: the outermost epidermis, the mid-layer dermis, and the underlying subcutaneous fat; these layers are composed of keratinocytes, immune cells, corneocytes, melanocytes, and microbiome communities (27, 28). In addition to the structural non-immune cells, such as endothelial cells and fibroblasts that contribute to collagen synthesis and structural integrity of the skin in the steady state, the skin-resident immune cells (macrophages, mast cells, and B and T lymphocytes) are involved in the maintenance of tissue homeostasis of the healthy skin (28).

In the absence of any skin injury, epidermal Langerhans cells (LC) and papillary dermal dendritic cells (DCs) control skin commensal-specific T cells and the presentation of environmental self-antigens. LCs regulate neutrophil influx and bacteria invasion (29). Dermal macrophages, which require the cytokine IL-34 for differentiation and self-renewal [32] and are mainly derived from monocytes, participate in the birth and maintenance of hair follicles, phagocytosis of pathogenic cellular debris, and the dispensable recruitment of peripheral immune cells (30). Lymphocytes can be found in both the epidermis and the dermis. αβ T lymphocytes localize near the hair follicles (HF) bulge to prevent autoimmunity, control the microbiome, and recruit other lymphocytes to the skin. A recent study on human prenatal skin development highlights the critical roles of Tregs and innate lymphoid cells as key modulators of the local immune environment that supports HF development (20, 31). Recently, the function of mast cells in cutaneous homeostasis has been unraveled, revealing that mast cells (MCs) play a crucial role in maintaining the epidermal barrier function of the skin. Skin mast cells are shown to exhibit tolerance to commensal bacteria through their interactions with dermal fibroblasts (32). Due to their low abundance and dispensability in skin hemostasis, neutrophils are not considered skin-resident immune cells.

Following a challenge to the skin integrity, the immune response is central in the wound healing process. Briefly, the functions of these skin-resident immune cells can be described in the following wound-healing stages:

### 2.1 Hemostasis

A wound, which can be either acute or chronic depending on the type of healing mechanism involved, is created when the standard tissue architecture is disrupted (33). Immediately after tissue integrity compromise, hemostasis begins. While platelets are the primary players in stopping bleeding and facilitating clot formation, immune cells also play a role in this initial phase of wound healing. Platelets, through the engagement of different receptors, cleave into components of the damaged extracellular matrix and form an aggregate, sealing off the injured area. Platelets secrete growth factors and chemoattractants, such as platelet-derived growth factor (PDGF) and transforming growth factor-beta (TGF-β), which are necessary for stimulating tissue repair (34). MCs influence the dermal dendrocyte expression of factor XIII by releasing TNF-α. Recruited by anaphylatoxins C3a and C5a, MCs are involved in hemostasis through protease and protease inhibitor level regulation (35).

### 2.2 Inflammatory phase

Known as first responders at injury sites within the first three days, neutrophils form neutrophil extracellular traps (NETs) by releasing pro-inflammatory cytokines, chemokine IL-8, lipid mediator chemoattractants such as leukotriene B4, and bactericidal peptides, thereby clearing pathogens. These secreted factors and receptors facilitate the recruitment of additional neutrophils from the circulation into the wound bed (36, 37). Early studies on neutrophil depletion in normal wounds, aimed at deciphering their roles, have suggested that neutrophils appear not to have a significant effect, but are essential in infected wounds. These findings may be related to their central role in wound infection clearance (38). Abnormal activities of neutrophils can be detrimental to wound healing.

Shortly after neutrophils, circulating monocytes, which can exist transiently in the tissue, infiltrate the wound from the periphery and differentiate into macrophages and DCs in response to the inflammatory environment. Before some of them undergo apoptosis, they secrete pro-inflammatory cytokines and chemokines (39). Macrophages, similar in characteristics to neutrophils, respond to damage-associated molecular patterns (DAMPs), secrete pro-inflammatory cytokines such as TNF-α, IL-1, IL-6, ROS, and nitric oxide (NO), and orchestrate the immune response of clearance of pathogens and germs, wound debris, and apoptotic cells (40). These cytokines, driven by the pro-inflammatory M1 macrophages, recruit other cells, including helper T cells, epithelial cells, and fibroblasts, to the inflammatory immunological wound surface (41, 42).

MCs can infiltrate the wound site through the monocyte chemoattractant protein (MCP-1) released by macrophages and keratinocytes (43, 44). They release mediators such as histamine, VEGF, IL-6, and IL-8, which increase endothelial permeability, promote vasodilation, and facilitate the migration of inflammatory cells, including monocytes and neutrophils, to the injury site (45).

DCs connect the innate and adaptive immune system. DCs express pattern-recognition receptors that allow them to respond to tissue damage or infection-related antigens, migrate to lymph nodes close to the tissue where they present the processed antigens, and stimulate T cells via antigen-MHC-T cell receptor complex (46). Following the presentation of antigens by DCs, naïve CD8+ T cells differentiate into various subsets of T cells, including Th1, Th2, and Th17 cytotoxic effector T cells, as well as memory CD8+ T cells that are activated upon secondary exposure (47). In addition to the previously mentioned effector cells, CD4+ Tregs exist that, rather than fueling inflammation, regulate the intensity of the immune response and help prevent reactions against self-antigens (48). The comprehensive impact of Tregs in cutaneous wound healing is discussed further in this review.

### 2.3 Proliferative phase

For wound healing to occur, these pro-inflammatory macrophages must transition into the anti-inflammatory M2 phenotype during the subsequent key stage, the proliferative stage (49). This third phase of wound healing is characterized by the resolution of inflammation, formation of granulation tissue, angiogenesis, and re-epithelialization (50). M2 macrophages primarily secrete anti-inflammatory and pro-angiogenic mediators, including arginase-1 (Arg-1), IL-10, transforming growth factor-β1 (TGF-β1), and vascular endothelial growth factor (VEGF). This M1 to M2 macrophage mediator switch facilitates the prompt resolution of inflammation and promotes angiogenesis (51). Collagen is deposited by endothelial cells, keratinocytes, and fibroblasts, creating a transient extracellular matrix. Growth factors generated by M2 macrophages promote angiogenesis, the process by which endothelial cells build new blood vessels (52).

DCs also help maintain the balance between pro-inflammatory and anti-inflammatory signals by secreting cytokines and recruiting fibroblasts, which play a crucial role in collagen deposition and tissue repair (53). Endogenous stimulating factors, such as granulocyte-macrophage colony-stimulating factor (GM-CSF), VEGF, IL-33, IL-7, and IL-3, attract basophils, a less abundant type of granulocyte (54), which secrete IL-4 to facilitate fibroblast proliferation and collagen synthesis (55). Through their interactions with keratinocytes and fibroblasts, MCs have been shown to induce fibroblast proliferation via IL-4, VEGF, and basic fibroblast growth factor (bFGF) (35).

### 2.4 Remodeling phase

This last phase constitutes wound contraction caused by the differentiation of fibroblasts into myofibroblasts, degradation of the ECM by matrix metalloproteinases (MMP) and tissue inhibitors of metalloproteinases (TIMPs), replacement of type III collagen with stronger type I collagen fibers, regression of angiogenesis, and simultaneous apoptosis of vascular cells (56). The remodeling phase has the relatively longest completion time.

Innate immunity is well appreciated for its role in wound healing. While lymphocytes are present in damaged skin, prior studies on athymic nude mice and fetal skin, characteristic of the absence of T cells and low lymphocytes, respectively, demonstrated rapid wound healing, suggesting that T lymphocytes may not be essential or sufficient for wound healing (57). A large fragment of research has followed that observation to explore the roles of T cells in tissue repair. T lymphocytes are essential for the resolution of dermal scarring (58, 59). Recent reports indicate that Tregs infiltrate wounds and modulate the regenerative response. That is particularly discussed in this review.

## 3 Skin Tregs and their origin

Tregs are adaptive immune cells that express the transcription factor Forkhead box P3 (Foxp3), the significant master transcription factor, and reduce overactive immune responses after recognizing diverse self and foreign antigens (60). As the primary transcription factor targeting Foxp3 stability, expression, and function, targeting Foxp3 directly equals targeting Tregs. Foxp3 is an underlying target in many physiological conditions, and its modification at different levels has been reviewed elsewhere (61, 62). Originally referred to as ‘suppressor T cells’, numerous studies have demonstrated that Tregs inhibit autoimmune tissue injury (63), facilitate organ transplantation (64) and wound healing (65), and possess detrimental roles in tumor biology (66). Although several reviews have discussed the maintenance role of Tregs in other tissues (67), this review specifically summarizes recent reports that converge on Treg cell-mediated cutaneous repair in the context of acute and diabetic wound healing. Additionally, the mechanisms of action of skin Tregs and their interactions with other cells are highlighted. In detail, we elucidate the identification of a distinct phenotype of ‘repair’ or ‘tissue healing’ Tregs in skin settings and propose potentially reliable immunotherapeutic tools involved in treating cutaneous acute and non-healing chronic wounds.

Tregs arise from two primary sources: thymic-derived Treg (tTreg) cells, which develop in the thymus upon interaction with self-antigens, and peripheral Treg (pTreg) cells, which differentiate from naïve T cells in response to tolerogenic stimuli. While tTregs are stable in their regulatory function and FOXP3 expression, pTregs can vary in their suppressive capacity based on environmental cues. Tregs can be further classified into induced Treg (iTreg) cells when generated *in vitro*. Notably, tTregs recognize self-antigens, whereas pTregs are more responsive to non-self-antigens (68, 69). Tissue-resident Tregs with distinct phenotypes have been identified in various locations, including skeletal muscle, skin, colon, cardiac muscle, lungs, liver, and the central nervous system (CNS) (47).

**Figure 1:**
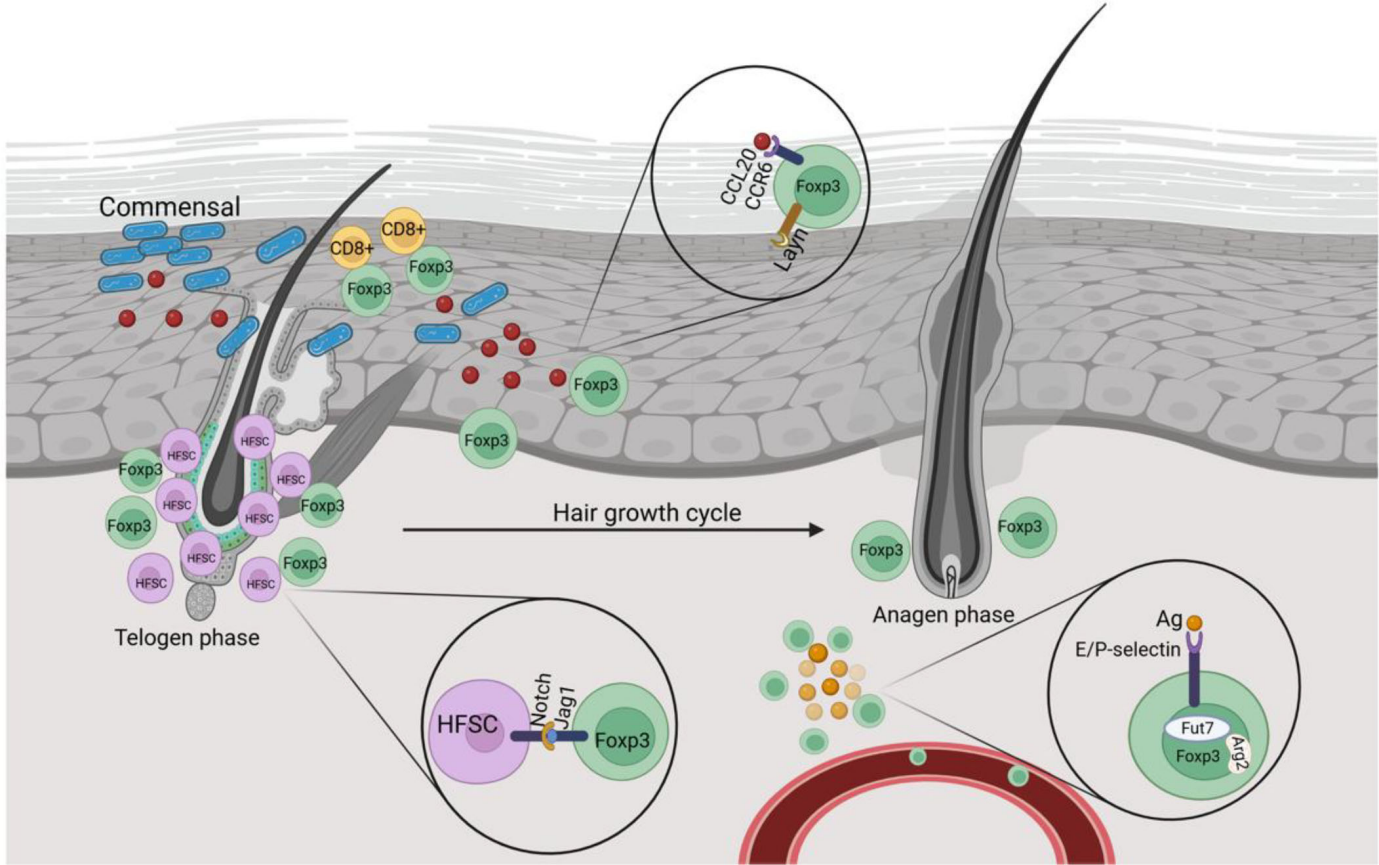
Localization and conceptual roles of cutaneous skin Tregs in homeostasis and hair regeneration. Schematic representation of the localization of cutaneous Tregs to the epidermis, hair follicles, and CD8^+^ T cells during postnatal development, their role in commensal tolerance, and adaptation during the hair growth cycle. Created at https://BioRender.com.

Murine skin CD4^+^ T cells seem to have a slightly higher population of Tregs than in humans. Out of the total CD4^+^ T cells, 30–50% and 20–30% are Tregs in mouse and human, respectively (70, 71). The absence of specific information complicates the identification of the origin of cutaneous Tregs. However, in a scenario of bacterial colonization in mice’s skin, a surge of activated Tregs was shown to fill the skin during the early neonatal period. Furthermore, inhibiting the lymphoid emigration of T-cells by treatment with FTY720, a sphingosine-1-phosphate receptor antagonist, resulted in the preferential concentration of Tregs in the thymus rather than in skin-draining lymph nodes, indicating that the thymus may be the origin of Treg migration (72). To establish commensal-immune tolerance, skin Tregs migrate to the epidermis and neutralize CD8+ T cells activated by the influx of commensals, which can spike inflammation in the tissue (73) (**Figure 1**).

Characterization of human Tregs from healthy tissues and their roles, as revealed by single-cell chromatin accessibility research, has shown a prevalent tissue repair gene signature associated with Tregs that express the transcription factor BATF and the skin-homing receptor CCR8, in addition to core markers such as CTLA-4, CD25, and Foxp3 (74). In homeostatic conditions, the expression of CCR10 enables the function and balance of Treg-Teff cells in skin-resident immune cells (75).

Previous observations indicate that most Tregs in the skin predominantly localize to hair follicles and also suggest that most skin Tregs feature a memory phenotype, similar to that of memory T cells (CD45RO), and are dermis-enriched (71). However, a specific analysis of the skin of Foxp3-GFP mice demonstrated that a minor proportion of Foxp3+ cells were found in other anatomically distinct regions of the skin, primarily the interfollicular epidermis (IFE) (76). Perhaps these IFE-Tregs are responsible for tissue regeneration in cases of minor damage to the stratum corneum.

Tissue homeostasis or regeneration is regulated not only by Tregs originally posited at the wounded tissue, but also by the recruitment of Tregs from the periphery to injury sites. However, when inflammation is provoked in the skin, plasmacytoid DCs (pDCs) accumulate in the skin wound (77), process peripheral antigens, and transport them to the thymus and lymph nodes to induce immune tolerance (78, 79). Through this means, commensal antigen-specific Tregs could be generated, tracing the origin of skin Tregs to the thymus. In summary, skin Tregs can be categorized into two types: skin-resident Tregs (those that accumulate in the skin during the neonatal period) and migratory skin Tregs (those that migrate into the skin after an injury) (80).

In an attempt to determine the adhesion molecules selectively used by skin Tregs, Mehta and colleagues speculate that Tregs can adhere to tissues to enhance interactions with surrounding local cells. Through an ECM-binding C-type lectin receptor highly expressed in human and mouse Tregs, called Layilin (Layn), Tregs anchor on the skin, enhancing their accumulation (81). An earlier report revealed that cutaneous Tregs express cutaneous lymphocyte antigen (CLA), homing receptor CCR4, and adhesion molecule integrin αvβ8 (76). Elsewhere, CCL17 is mainly expressed by endothelial cells and CCL22 by dermal DCs in inflamed skin, which are recognized chemokine ligands for CCR4 (82). These molecules are sequentially involved in regulating T-cell homing to the skin, where CCL17 promotes vascular permeability and immune cell recruitment, and CCL22, which appears to have greater dominance in inducing CCR4, guides subsequent migration in the skin (83). Healthy human skin Tregs have been reported to preferentially express TNFRSF9 and a mitochondria-localized enzyme, arginine-2 (Arg2), which allows tissue adaptation and function through mTOR signaling (84).

Evidence suggests that Tregs imitate effector target cells by expressing their transcription factors, such as T-bet for Th1 and interferon regulatory factor-4 (IRF-4) for Th2 effector cells (85). Over 70% of skin Tregs preferentially express GATA3 during homeostasis; however, its deletion does not influence Treg profile or induce overt skin-related phenotype (86). An epigenetic landscape study of the similarities between different tissue Tregs revealed that skin Tregs and Fat Tregs are Th2-polarized, indicating the presence of commonly shared Th2-biased subsets of tissue Treg ST2 cells (87).

## 4 Tregs in cutaneous acute wound healing

Acute wound healing consists of four overlapping phases: hemostasis, inflammation, proliferative, and remodeling. Moderate immunomodulation is crucial in wound healing, as processes that regulate macrophage polarization and cytokine production are integral to wound-healing research (25). An example of immune regulation is the modulation of macrophages through mechanisms including enhancing macrophage efferocytosis, inhibiting their recruitment, and regulating their polarization (88). Tregs are involved in the latter stages of wound healing. Elucidating the response to self-antigen by Tregs in tissues, inflammation initiated by OVA secretion in a mouse epidermis-inducible model was not resolved by conventional T cells. Instead, a further antigen expression caused a short involvement of memory skin CD25^low^KLRG1^high^ CTLA4^high^CD127^high^ Tregs (89).

Tregs serve an essential function in regulating skin homeostasis through both direct and indirect approaches, utilizing their anti-inflammatory and anti-apoptotic properties (90). Their secretion of anti-inflammatory cytokines (such as TGF-β1 and IL-10) promotes the polarization of anti-inflammatory macrophages and inhibits the inflammatory response (91), modulating the amplitude of the immune response and inhibiting an immunological reactivity towards self-antigen (48). In comparison to wild-type controls, wound healing is delayed in Treg-depleted mice in a study that employed Foxp3-DTR transgenic mice, in which Tregs are depleted after injection of diphtheria toxin (65). Investigating the presence and infiltration of skin Tregs in the early stage after injury, Yue-wen and colleagues demonstrated that skin Tregs promote wound healing after skin burns, and the frequency of skin Tregs markedly decreases after the early stage (92). In both wild-type and activin-transgenic mice, depletion of Tregs resulted in overexpression of IFN-γ, IL-17A, and IL-4, along with potentially other cytokines. Inadequate vessel formation, diminished contraction, and hindered re-epithelialization were also reported (93).

Recently, a study employed single-cell RNA sequencing (scRNA) and immunofluorescent imaging to functionally distinguish the distinct T cell subsets involved during the inflammatory phase of wound healing (94). In addition to the isolated activated T cells, cytotoxic T lymphocytes (CTLs), and exhausting T cells responsible for activation of NFκB signaling, and Th17 cell differentiation, inducing NK cell-mediated cytotoxicity, promoting PD-L1 expression and interfering with Th1/Th2 differentiation, respectively, further analyses revealed that they are primarily involved in cytokine-receptor interactions and the inhibition of Th17 cell differentiation (94). The validation of exhausting T cells in the inflammatory phase by this study demands further research.

Skin Tregs have been implicated in the re-epithelialization stage. Tregs’ conditioned media secrete several cytokines, such as IL-8 and IL-10, and increase the expression of MMP-1, stimulating the epithelial-mesenchymal transition (EMT) of HaCaT keratinocytes (95). To unravel whether there is any difference in immune microenvironment between small and large wounds with implanted scaffold, a high-dimensional multiomics study revealed that while γδT cell was the primary subtype of T cells in minor wounds, there was a greater frequency in large wounds, suppressing excessive deposition of collagen and aiding the differentiation and migration of HFSCs (96).

**Figure 2:**
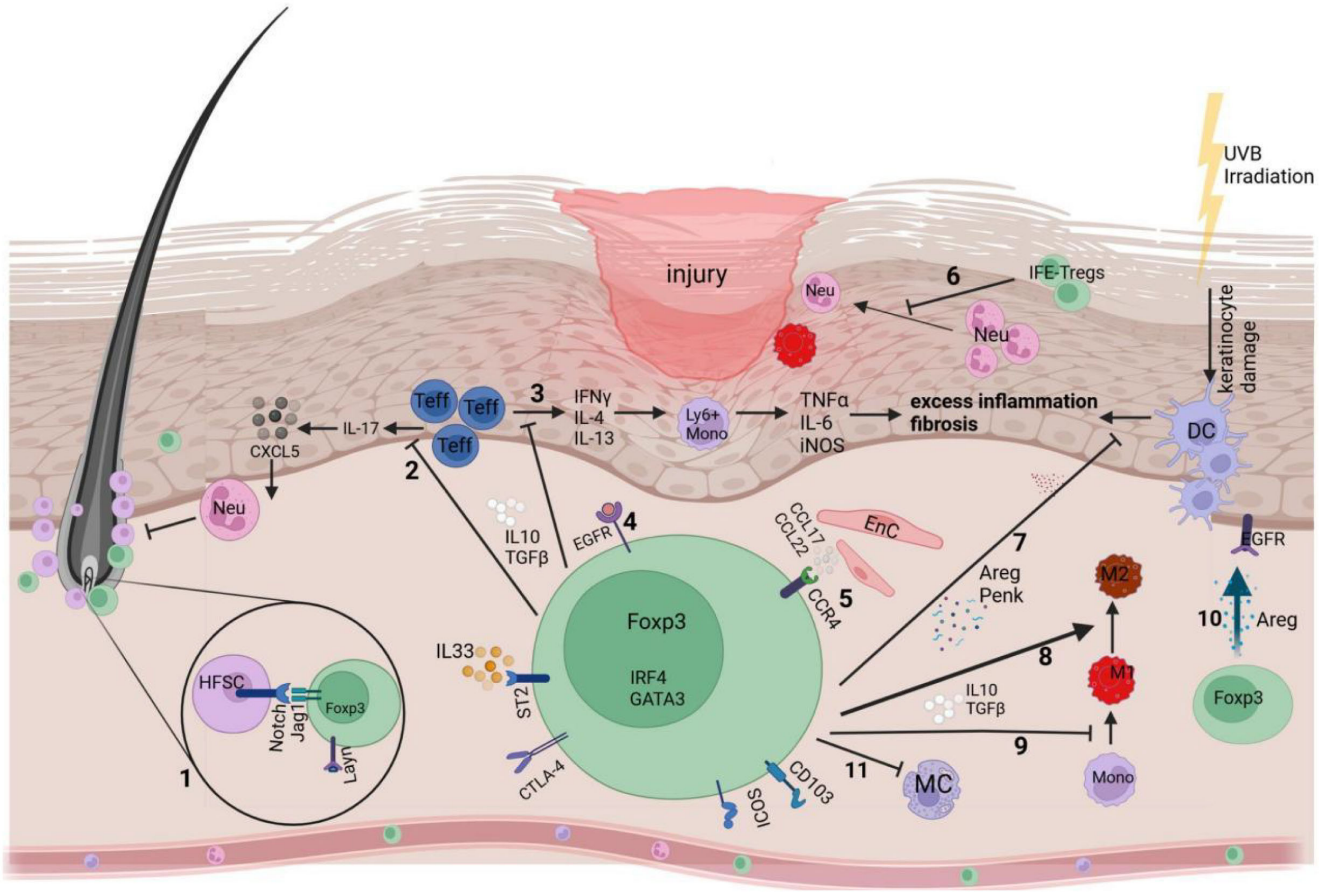
Skin Tregs promote acute skin repair. Tregs promote acute skin repair through multiple mechanisms. 1) Through Notch-Jagged1 signaling, Tregs enhance stem cell differentiation. 2) Tregs also express skin-anchoring LAG-2, which enables them to repress the accumulation of Neutrophils. 3) Tregs suppress Teff cell secretion of pro-inflammatory cytokines. 4) Tregs utilize the EGFR pathway to repair injury. 5) Tregs interact with CCL17 expressed on endothelial cells. 6) Tregs suppress the neutrophil influx into the wound bed and neutrophil-driven cytokine release. 7) CD103+ dendritic cells (DCs) induced by UVB light tend to cause inflammation but are suppressed by Tregs. 8) Tregs promote the conversion of M1 to M2. 9) Tregs suppress the differentiation of monocytes to pro-inflammatory macrophages. 10) Tregs secrete Areg that interacts with epithelial cells. 11) Tregs interact with Mast cells to prevent excess degranulation. IFE, Interfollicular epidermis; Jag1, Jagged 1; M1, Pro-inflammatory macrophages; M2, anti-inflammatory macrophages; Mono, Monocytes; Neu, Neutrophils; CTLA-4, Cytotoxic T-lymphocyte-associated protein 4; Jag1, Jagged 1; CCL, CC chemokine ligand type; CCR, CC chemokine receptor type; CXCL, cysteine X cysteine ligand; Areg, Amphiregulin. Created at https://BioRender.com.

These data shed light on the role of Tregs, provide insight into the mechanisms of wound repair, and suggest a potential application of Treg-based cell therapy through topical administration (**Figure 2**). Researchers continued to explore the possibility of this strategy. Until now, the exact mechanisms of Tregs’ action in cutaneous wound healing are incomplete. However, despite reports of the controversial involvement of T cells in wound healing, multiple pre-clinical studies unequivocally demonstrate the key role Tregs play in the healing of cutaneous wounds. These beneficial roles facilitated by Tregs in acute wound healing are tightly regulated; however, in the context of diabetes, they are significantly disrupted, contributing to the pathophysiology of many chronic wounds.

## 5 Role of Tregs in chronic diabetic wounds pathophysiology

**Figure 3:**
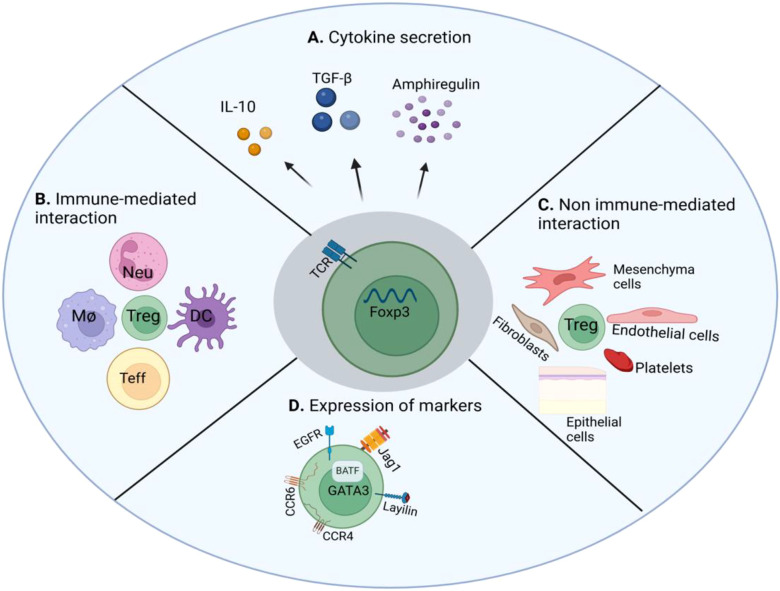
The mechanisms of skin Tregs in tissue repair. **(A)** Tregs can secrete cytokines IL-10 and TGF-β to suppress effector T cells and pro-inflammatory macrophages, and express Areg to promote the growth of keratinocytes. **(B)** Tregs can interact with Neutrophil, Macrophages, and Dendritic cells to regulate their immune functions. **(C)** Tregs can influence the migration and wound-healing properties of mesenchymal cells, fibroblasts, endothelial cells, and epithelial cells. **(D)** Tregs can express Jag1 and EGFR to facilitate hair follicle regeneration and stem cell functions. Treg cell, Regulatory T cell; IL-10, Interleukin-10; TGF-β, Transforming Growth Factor-β; Neu, Neutrophil; DC, Dendritic cell; Mϕ, Macrophage. Created at https://BioRender.com.

Acute wounds undergo a sequence of molecular processes that restore the tissue’s structural integrity, whereas chronic wounds do not heal effectively. The healing process is impeded and characterized by pathological processes, including ongoing inflammation, persistent infections, tissue necrosis, defective re-epithelialization, decreased angiogenesis, and overproduction of reactive oxygen species (**Figure 3**) (25). Chronic wound healing is particularly impaired in diabetes, increasing the risk of complications and infections. The intricate interplay between pathophysiological factors and Tregs is critical in promoting diabetic wound healing. Recent investigations have highlighted the involvement of Tregs in modulating these factors, emphasizing their importance in chronic diabetic wounds. People with diabetes with hyperglycemia have chronically high blood glucose levels. This syndrome may damage blood vessels, limiting blood supply to skin injuries and immune cell activity (97). Hyperglycemia lowers ANGPTL4, an anti-inflammatory stromal cell protein. It also affects Treg recruitment and function by increasing serum pro-inflammatory cytokines like monocyte chemoattractant protein-1 (MCP-1) and MMPs and disrupting Treg-immune cell interactions (98–100). Excessive inflammation negatively affects healing in diabetic wounds. Specifically, IL-1β, TNF-α, and MCP-1, which are elevated in the blood and abnormal, contribute to this issue. Diabetic wounds often exhibit persistent inflammation due to the presence of macrophages and neutrophils (101). Long known for their anti-inflammatory properties, Tregs modulate the dysregulated pro-inflammatory cytokines in chronic wounds.

Hypoxia, or low oxygen supply, can impair cellular activities, including monocyte wound healing. HIF-1 levels are maintained by hypoxia. Hypoxic conditions impair Treg function, stability, and the balance between ROS and antioxidants. Chronic HIF-1α activation in diabetic wounds can destabilize Tregs and turn them into pro-inflammatory Th17 cells (102). Local restoration of miR-210 enhances diabetic wound healing, suggesting modulation of the aberrant HIF-1α signaling pathway (103, 104). Hyperglycemia affects HIF-1α signaling in diabetic foot ulcers, with varying stage-specific effects (105). Hyperglycemia reduces HIF-1α stability and Tregs, suggesting a link between hypoxia and Treg activities in diabetic wounds (103).

Diabetic wound beds have decreased angiogenesis due to persistent inflammation, endothelial dysfunction, and hyperglycemia-induced cellular damage. Tregs play a tissue- and disease-specific role in angiogenesis (106). Numerous studies have linked VEGF signaling to the survival and proliferation of intratumoral Tregs in the tumor microenvironment. Without VEGF, Tregs can indirectly influence angiogenesis by affecting the function of immune cells. In diabetic mice, CD4+ Th1 cells and Tregs had opposing effects (107). Recent research shows that Tregs boost angiogenesis in diabetic mice skin wound models.

Diabetic complications include neuropathy. Tregs appear to affect peripheral nerve regeneration, especially diabetic neuropathy nerves. Tregs correct neuron-immune communication dysfunction in Schwann cells to enhance nerve regeneration, delaying nerve restoration and reducing wound site sensory input (108). Diabetic neuropathy inhibits neurotrophic signaling, slowing wound healing. Substance P (SP) and calcitonin gene-related peptide (CGRP) are neuropeptides that regulate immune cell responses in the skin and are associated with diabetic wound healing. Tissue restoration requires a balance of pro- and anti-inflammatory neuropeptides (109). Tregs regulate neuropeptide-secreting Schwann cells. Neuropeptides, like Tregs, can regulate diabetic wounds by reducing the inflammatory phase, notably in diabetic foot ulcers (110).

Most chronic wounds affect older people. Higher amounts of inflammatory cytokines in aged tissues can hinder Treg-mediated regeneration. With aging, the function of the immune system declines, and extrathymic induction of Foxp3 is impaired (111). Additionally, cell-autonomous repair skills are compromised (112). These pathophysiological considerations suggest that diabetic tissue healing alters the function of Tregs. Context-specific research is necessary to elucidate the importance of Tregs in diabetic wound healing.

## 6 Mechanisms of action of skin Tregs in tissue repair

Generally, Tregs demonstrate immune tolerance and suppression through various mechanisms due to their heterogeneity. These include the secretion of anti-inflammatory factors such as interleukins IL-10 and IL-35, transforming growth factor-β (TGF-β); the suppression of the release of tumor necrosis factor-α (TNF-α) interferon (IFN)-γ, and IL-6; apoptosis of target cells; and the inhibition of T-cell activity by impeding nutrient availability and proliferation via cytotoxic T-lymphocyte antigen-4 (CTLA-4) (113, 114). In the context of tissue repair, the mechanisms through which Tregs facilitate wound healing can be summarized to include the following (**Table 1**):

**Table 1:** The impact of skin Tregs on tissue repair.

Impact of Tregs	Mechanisms and mediators	Treg deficiency/dysfunction	References
Inflammation resolution	Suppress Th1/Th17 cytokines Promote the synthesis of anti-inflammatory cytokines, such as IL-10 and TGF-β. Promote M2 macrophage polarization.	Impaired phase transition due to prolonged inflammation Destruction of tissues	(65, 70)
Promote angiogenesis	Regulate pro-angiogenic factors (e.g., VEGF) Support endothelial cell function.	Impaired vascular repair due to inadequate angiogenic signals possible recruitment of dysfunctional Treg subsets Hypoxia/Ischemia	(93, 115)
Support re-epithelialization & matrix remodeling	Signal amphiregulin/EGFR to keratinocytes interaction with fibroblasts modulation of MMP/TIMP balance	Halt to tissue regeneration Dysregulated/weak ECM Non-migrating epithelium	(116)
Immune tolerance and microbiome balance	Control effector CD8+ T cells establishment of commensal tolerance acute recruitment of inflammatory T cells	Loss of immune homeostasis Increase in risk of infection Dysbiosis	(117)
Tissue adaptation	Expression of chemokine receptor (e.g., CCR4, CCR2) expression of tissue-resident phenotype (e.g., Jagged1, Arg2) Suppress responses that lead to pro-fibrotic gene expression	Impaired chemotaxis and Treg recruitment Failure to adopt a wound-specific phenotype	(86, 118)

### 6.1 Cytokine secretion

Tregs secrete immunomodulatory cytokines such as IL-10 and TGF-β, crucial for reducing inflammation and promoting healing. IL-10, in particular, has been shown to enhance the anti-inflammatory response and facilitate macrophage polarization towards a pro-healing phenotype, inducing neutrophil apoptosis in the process (119). The interaction of a positive feedback regulation between IL-10 and Epidermal Growth Factor Receptor (EGFR) suggests that increased levels of IL-10 can enhance EGFR activity, which in turn may further elevate IL-10 levels (120). TGF-β1 signaling is crucial for Treg development globally in the thymus and non-lymphoid tissues. Through TGF-β signaling of skin Tregs, reports have shown that TGF-β drives hair regeneration (121) and promotes epithelial barrier repair (76). Amphiregulin (Areg) is an EGFR ligand but can also be referred to as a cytokine in a tissue repair context. Tregs secrete Areg, which binds to the receptor expressed on Tregs in a paracrine signaling manner. While the complete mechanism by which Areg contributes to direct wound healing in tissues, including muscles and lungs, is yet to be determined, it has been previously demonstrated that Areg increases the suppressive function of both *in vivo* and *in vitro* Tregs (122). A recent study demonstrated that Treg-derived Areg plays a role in epithelial tissue maintenance and repair beyond the skin; in the thymus, Areg boosted thymic regeneration following injury (123).

**Figure 4:**
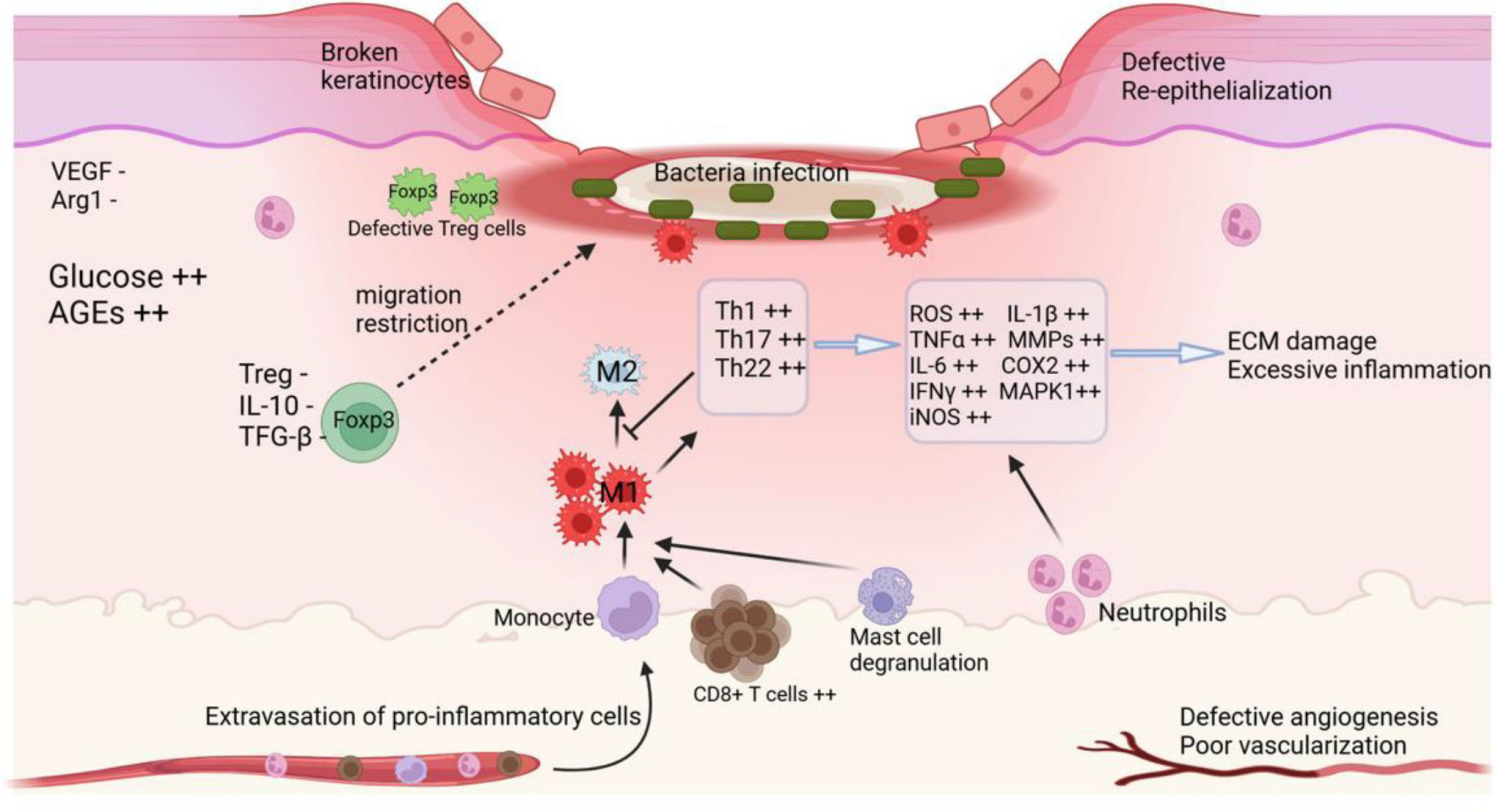
A typical diabetic chronic wound. Diabetic chronic wounds are characterized by recurrent bacterial infections, prolonged presence of inflammatory cells, including neutrophils and M1 macrophages, high level of pro-inflammatory T cell subtypes such as CD8+ T cells, Th1, Th17 and Th22; hypoxia; increased ROS; hyperglycemia; defective angiogenesis, re-epithelialization, and vascularization; secretion of MMPs by keratinocytes; ECM damage; and the restriction of Treg migration. Even when present, they appear dysfunctional. Created at https://BioRender.com.

Upon tissue damage, multiple mediators have been linked to tissue Tregs (**Figure 4**). In other tissues, such as muscles and brains, interleukins IL-18 and IL-33 have been linked to the proliferation of Tregs and injury repair (124). Some tissue-damage mediators and basal antigen stimulation have been reported to trigger Treg actions in skin injury. Glucocorticoids are one of the components that signal repair in the skin. Signaling mediated by the glucocorticoid-TGF-β3 axis, Foxp3 interacts with HFSCs to support tissue regeneration and hair follicle proliferation. Through the activation of SMAD2/3 by glucocorticoid receptor signaling, the repair is facilitated (121). These findings indicate that early signals from tissue injury trigger Treg-mediated tissue repair. Niche-specific factors and antigen stimulations likely also play a role (125). Characterizing the cells that generate these signals would contribute immensely to this area of skin-specific tissue repair research.

Another signaling component is the ultraviolet B (UVB) irradiation. Upon UVB irradiation, Tregs produce opioid precursor proenkephalin (Penk) and Areg to promote keratinocyte outgrowth, suggesting a healing function of UVB-skin Tregs. CD103^+^ DCs induced by UVB light tend to cause inflammation by encapsulating self-mRNAs induced by damaged keratinocytes, but Tregs suppress these damage-causing DCs (126). Mechanistically, Penk^+^ Treg-derived enkephalins signal endothelial cells via the δ-opioid receptor (δ-OR), thereby promoting angiogenesis and healing through neuro-immune signaling (127).

### 6.2 Interaction with other cells

In addition to secreting cytokines, Tregs also carry out their reparative functions by interacting with other cells in the tissue through both immune-based and non-immunological mechanisms. Through their specific phenotype, which regulates undesirable immune activities primarily mediated by other immune cells, skin cells create an anti-inflammatory and anti-apoptotic environment within the tissue. This activity facilitates the repair and regeneration processes either directly or indirectly (90).

#### 6.2.1 Treg-neutrophils dynamics

Tregs can directly influence neutrophil behavior by promoting their secretion of anti-inflammatory mediators. This interaction helps mitigate the damaging effects of neutrophils during the inflammatory phase of wound healing (21). Lipopolysaccharide-activated Tregs inhibit the inflammatory activity of neutrophils by inducing heme oxygenase-1 and suppressor of cytokine signaling 3 (SOCS3), and by inhibiting the production of IL-6 *in vitro* (128). Surprisingly, Jag1^+^ Tregs also promote the retention of neutrophils in the skin following skin injury (115). Hence, neutrophils facilitate wound healing through mechanisms that include the dissipation of infiltrating pathogens, debris, and damaged cells (129).

#### 6.2.2 Treg-macrophages dynamics

Tregs inhibit macrophage recruitment and the secretion of TNF-α and IL-6 (117). They influence the polarization of macrophages towards the M2 phenotype, which is associated with anti-inflammatory and tissue repair functions. They achieve this by secreting cytokines such as IL-10, which promotes M2 polarization and inhibits pro-inflammatory M1 macrophage activation (21, 65).

#### 6.2.3 Treg-dendritic cells dynamics

Through direct contact and cytokine signaling, Tregs can induce a tolerogenic phenotype in DCs. This process involves downregulating co-stimulatory molecules (like CD80/CD86) on DCs, which reduces their ability to activate naïve T cells. This interaction helps to limit the inflammatory response during the early phases of wound healing. Treg-DC interaction skewed CD4^+^ naïve T cell polarization toward a regulatory phenotype, negatively affecting their maturation and function (130).

#### 6.2.4 Treg-mesenchymal cells dynamics

Since Growth arrest-specific (Gas) 6 has been studied to enhance the suppressive function of CD4^+^CD25^+^ Tregs mainly through a TAM receptor (131), and that Treg-derived Areg promotes muscle-associated mesenchymal stem cell differentiation to facilitate muscle repair (132), it is possible to speculate that there is a possible Treg-mesenchymal cell interaction in the skin.

#### 6.2.5 Treg-epithelial cells dynamics

Data obtained following the skin barrier breach demonstrate that dermal and epidermal Tregs are broadly similar, cooperatively and preferentially expressing transcriptional pathways relevant to epithelial cell biology. Through the expression of integrin αvβ8, Tregs act directly on epithelial cells (76). Keratinocytes are the primary skin parenchyma cells that comprise most of the epidermis and play a crucial role in wound healing. HFSCs are indispensable in the study of Treg and epithelial cells. Studying the Treg-keratinocyte signaling axis, *Mathur* et al. (2019) used a subacute skin injury model to highlight a mechanism by which Tregs promoted an alternative fate decision for HFSCs, usually poised for hair generation, to differentiate into stratified epithelium necessary for skin barrier repair. In this model, Tregs repressed CXCL5 production by keratinocytes, thereby limiting the recruitment of inflammatory Th17 cells and neutrophils, which promotes healing (116).

In a study that utilized models of hair regrowth to investigate Treg cell interactions with hair follicle epithelial stem cells (HFSCs), tissue-resident Tregs of an activated phenotype were enriched during the anagen (follicle regrowth) phase of hair growth compared with the telogen (follicle quiescence) phase. Tregs localize to the HFSC niche to promote their differentiation during hair follicle cycling (70), influencing their function (116). A recent report explained how stem cells are preserved when they leave their niche for repair at the wound bed. The expression of CD80 on HFSCs is already present at the broader partial thickness wound site (133).

#### 6.2.6 Tregs-endothelial cells dynamics

These parenchyma cells line blood vessels and are responsible for angiogenesis, which involves the formation of new blood vessels. To explain the localization of CD4+CD25+Foxp3+ Tregs to both lymphoid and non-lymphoid tissues via the TCR, it was demonstrated that recognition of self-antigens expressed by endothelial cells in target tissues is instrumental for the efficient migration of Tregs *in vivo* (134). This supports previous reports that Tregs, via their disadvantaged TCR, are stimulated and egress into tissues through MHC-II-based antigen presentation by endothelial cells. While Treg transmigration is enhanced, other helper T cell subsets face the opposite fate. Recently, Treg-lymphatic endothelial cell interactions have been reported, clarifying the consequences of Treg transendothelial migration (TEM) (135). Since endothelial cells can express Thymus and Activation-Regulated Chemokine (TARC, also known as CCL17) during inflammation, the interaction between endothelial cells and CCR4-expressing Tregs is possible and warrants further study.

#### 6.2.7 Treg-fibroblast interaction

Evidence shows that Tregs and fibroblasts can engage directly through cell-surface receptors, such as PD-L1, on non-hematopoietic cells, including fibroblasts, which interact with PD-1 on Tregs. This PD-1/PD-L1 interaction helps maintain an anti-inflammatory environment, limiting fibroblast activation and reducing chronic inflammation in wound healing (136). Tregs also produce high levels of IL-10, which acts directly on fibroblasts. Tregs help ensure that the healing process proceeds efficiently without excessive tissue damage. A clear understanding of interactions between skin Tregs and tissue-resident non-immune cells could further lead to innovative therapeutic approaches for enhancing tissue repair in clinical settings.

#### 6.2.8 Treg-platelets interaction

The receptors on platelets and Tregs can facilitate a feasible direct interaction between the duo. As demonstrated in autoimmune systemic lupus erythematosus (SLE), P-selectin, expressed by platelets, can interact with the P-selectin ligand PSGL1 on Tregs (118, 137). Upon activation, platelet lysates release various derivatives, including CD40 ligand (CD40L), which is known to bind to and expand CD25+Foxp3+ Tregs, thereby releasing TGF-β (138). In summary, the interaction between Tregs and platelets in skin wound healing can be described as soluble factor-based rather than a receptor-ligand relationship.

### 6.3 Exclusive surface molecule expression

Several pre-clinical studies on wound healing have revealed that skin Tregs exclusively express specific molecules during cutaneous damage. EGFR and jagged-1 (Jag1) are common molecules that mediate skin tissue homeostasis and repair function.

#### 6.3.1 EGFR

Among other cytokines and growth factors, epidermal growth factor, through the EGFR pathway, invokes cytoprotection, mitogenesis, and migration in tissue repair (Bodnar, 2013). In an experiment examining gene expression levels in cells isolated from SDLN and skin, EGFR expression was measured in both Foxp3-positive and Foxp3-negative CD4^+^ T cells isolated from skin or skin-draining lymph nodes before wounding. However, no change occurred in dLNs, but a significant induction of EGFR expression was detected in skin Tregs following injury (65). The findings indicate that epidermal growth factor receptor (EGFR) expression is preferentially upregulated on Tregs within inflamed environments. It has consistently been reported that almost all EGFR^+^ cells were Foxp3^High^ and CD45RA^-^ (termed activated Tregs); these Tregs gained EGFR expression upon stimulation, as seen in the skin (65) and other tissues, such as muscles and lungs, after injury (132). Moreso, EGFR-positive Tregs exhibit more vigorous immunosuppressive activity than their EGFR-negative counterparts, as evidenced by increased immunosuppressive cytokine production, more potent inhibition of CD8^+^ T-cell proliferation *in vitro* (139), and enhanced Treg survival in inflamed tissues. These results indicate that EGFR and its ligands are necessary for Treg function in wound healing. Although the function of amphiregulin, one of the ligands of EGFR, on Tregs has been described in muscle and lung tissues, further studies are needed to elucidate its role in cutaneous injury.

#### 6.3.2 Jagged-1

Jag1 is a ligand of the Notch signaling pathway, important in the cellular communication of skin Tregs. In the context of tissue repair, Jag1 is highly expressed on the surface of skin Tregs in comparison to gene transcripts of skin-draining lymph node (SDLN) Tregs in response to inflammatory signals, distinguishing them from other immune cell types. Jag1, as a Treg mediator, interacts with Notch receptors on neighboring cells, influencing their differentiation and function (70, 115). However, Jag1 is dispensable in the homeostatic state of skin Tregs (115). The Jag1-Notch signaling axis enables Tregs to communicate with other cell types in the skin, such as DCs and keratinocytes, thus shaping the local immune landscape and ensuring a balanced response to environmental challenges (76, 121).

#### 6.3.3 Integrin αvβ8

Using transcriptomics research to identify the pathways involved in innate inflammation after skin injury, Moreau and colleagues identified that skin Tregs preferentially expressed integrin αvβ8 to influence epithelial cells (76).

#### 6.3.4 Layilin

Layilin is a C-type lectin-like transmembrane receptor expressed following a TCR-mediated activation in skin Tregs, influencing Treg adhesion, motility, and suppressive functions in the tissue (81).

#### 6.3.5 Chemokine receptors CCR4/CCR6/CCR8

For a sufficient amount of Tregs in the site of injury, skin-resident Tregs through the expression of homing receptors rely on CCR4-CCL17/22 (140), CCR6-CCL20 signaling (141), and chemokine CCR8 expression (74).

## 7 Contributions of skin Tregs in diabetic wounds

Complications from diabetic chronic wounds, otherwise known as diabetic cutaneous ulcers, significantly impact the quality of life and survival rates of patients (142). Among the multiple physiological and pathological factors that cause these symptoms, the continuous and excessive generation of inflammation, resulting from an imbalance between pro-inflammatory and anti-inflammatory signals, as well as reactive oxygen species (ROS), are key elements. Elevated levels of ROS lead to oxidative stress, further hindering the healing process by causing tissue damage (143). Excessive ROS and chronic inflammation activate MMP-9 expression through the NF-κB pathway, disrupting the balance of ECM deposition and remodeling and preventing epithelial closure (144). The destruction of the ECM, which provides a scaffold essential for cell migration, increases the risk of infection onset. Numerous strategies are being developed to eliminate excessive ROS production or suppress inflammatory responses in the diabetic wound bed (145). Many of these strategies have proved effective, but looking into the immune system might yield a better solution, especially for people with diabetes, who are characterized by multiple complications.

Diabetes, regardless of the type, has been linked to regulatory T cells; this could be either a fluctuation in frequency or an alteration in their functions. Recent studies have utilized cutting-edge technology to elucidate the cellular composition of the skin, thereby highlighting the distinct mechanisms employed by various T subsets during and before tissue repair (146). For example, Xu et al.’s findings validated a decline in the CD4/CD8 ratio and elevated levels of MAPK and inflammatory cytokines, which stimulate CD8+ T cells and IL-2 in DFU patients following keratinocyte infection (147). This ongoing inflammatory milieu creates a condition where regulatory functions are essential yet impaired.

The function of the adaptive immune system is little understood compared to the innate immune system’s role in exacerbating non-healing wounds, but several studies have explored the additional characteristics of Tregs. The exact mechanism of non-healing chronic wounds is complex and unclear. Aside from the secretion of cytokines, which is part of the mechanisms previously discussed in acute injury, independent of cell contact, Tregs can also secrete extracellular vesicles to exert their immune response regulation in autoimmune and inflammatory diseases (148). These Treg-derived extracellular vesicles are laden with microRNA cargo, which, upon delivery to recipient cells, suppresses effector T cells and influences antigen-presenting cell phenotypes (149).

Using different study models, the positive roles of Tregs in wound healing have been explored. While the functions of Tregs can be influenced by the severity of the disease and the wound microenvironment, they can still be beneficial in their contribution. Studies have shown that diabetic patients and animal models exhibit a significant reduction in CD4^+^CD25^+^FOXP3^+^ Tregs compared to their normoglycemic counterparts, which correlates with delayed wound healing. In this ischemic model, CD4^+^ blockade indirectly enhanced Treg function by decreasing inhibitory signals from effector T cells such as Th1 cells, thereby allowing them to exert their pro-angiogenic functions. Tregs, specifically secreting IL-10 and amphiregulin, contribute to the sprouting of angiogenesis in diabetic wounds by supporting the expression of pro-angiogenic factors, such as apelin, which is essential for endothelial cell function and the regeneration of the peripheral vascular system (107).

Diabetes mellitus patients possess systemic inflammation that may hinder Treg migration and promote the infiltration of inflammatory Th17 cells into wound tissue. Deficits in inflammation-suppressing Tregs can lead to persistent inflammation and non-healing wounds (150). This establishes a clear paradigm in which Treg deficiency is detrimental to diabetic wounds, and their restoration may be a therapeutic approach. Supporting this, subsequent research has focused on determining whether supplementary Tregs can play a role in tissue repair. A recent study demonstrated that local delivery of exogenous Tregs can enhance tissue healing in various models by rapidly adopting an injury-specific phenotype and modulating local immune responses. This approach could be particularly beneficial in chronic wound scenarios where endogenous Treg function may be compromised (21), consistently accelerating healing in preclinical models.

Similarly, treatment with cord blood-Treg-derived exosomes accelerated wound healing by suppressing inflammatory responses while enhancing angiogenesis and tissue remodeling in a diabetic mouse skin wound model (151). Furthermore, CCR2-engineered mesenchymal stem cell infusion reshaped the local microenvironment in diabetic wounds by inhibiting monocyte infiltration, remodeling the inflammatory properties of macrophages, and promoting Treg accumulation in response to the overexpression of CCL2, thereby accelerating tissue repair (152).

However, the narrative that more equals improved healing is complicated by contrasting evidence that suggests it impedes healing in some specific contexts. Critically, in some diabetic wound settings with a higher frequency of Tregs, these cells are largely unresponsive and dysfunctional and appear to contribute to the worsening of the injury (153). The enrichment of Tregs in diabetic wounds, as quantified by xCell-based deconvolution, may represent a failed compensatory response to the persistent local inflammation in the wound microenvironment (154). This crucial insight necessitates a deeper exploration of situations where the reparative capability of Tregs falter, and when Treg function could actually obstruct healing.

Initial inflammation is necessary for the clearance of bacteria. A group of researchers reported a negative impact of Tregs in diabetic mice, citing that Treg depletion supports the fending off of colonization and infection by bacteria to which it is continuously exposed. An ill-timed Treg response can prove detrimental to proper host defense (155). Later, the research team of Barros et al. utilized the ability of chemokine receptor CCR4, together with its ligands CCL17 and CCL22, to draw in and activate T cells in the skin. In that study, which employed a full-thickness diabetic murine wound model, it was shown that CCR4 hinders wound healing. CCR4 deficiency, as demonstrated by CCR4 knockout and antibody treatment, was associated with a decrease in Treg migration into injured skin, consistent with its function as a skin-homing receptor. Remarkably, diabetic wild-type mice demonstrated quicker wound healing than their untreated counterparts when neutralizing antibodies against CCR4 ligands (140).

This negative influence can be attributed to excessive accumulation at the inflammatory sites, thereby dampening inflammation that is necessary during the initial phase of wound healing. Therefore, for proper chronic wound healing, sufficient inflammation must be present to prevent hyper-inflammation during the initial inflammatory phase, but its activity must be regulated to allow for antimicrobial immune functions. For proper proliferation involving vascular angiogenesis to occur, the dysfunctional recruitment of must be avoided, as it could contribute to a delay in healing. Further research is necessary because there is little data on Treg’s involvement in diabetic and chronic wounds. This would help in drawing conclusions and influencing wound therapeutic approaches.

### 7.1 Impact of Tregs in the pathology of other chronic diseases

Over time, inappropriate wound healing, in addition to other factors, often triggers a wide variety of other chronic diseases, including other inflammatory diseases, cancer initiation, fibrosis, heart diseases, and arthritis (156). Exploring the role of Tregs in fibrosis, both acute and chronic depletion of GATA3^+^ Tregs in the skin resulted in activation of skin pro-fibrotic gene expression. Skin Tregs modulate Type 2 immune responses initiated by effector cells that drive fibrosis (86). Some limited successes have been reported in the application of Treg-secreting cytokines, including IL-10 and TGF-β, indicating the potential role of Tregs in fibrosis (157). Because fibrosis in tissues, such as liver fibrosis, carries a high risk of evolving into hepatocellular carcinoma (158), it is crucial to regulate the early stages of skin wound healing to prevent it from progressing to skin- and non-skin-related cancers. The involvement of Tregs in controlling early-stage inflammation during wound healing has already been studied. It is also important to note that, although epithelial cell proliferation, angiogenesis, and the dampening of inflammation are necessary for proper wound healing, uncontrolled healing responses can lead to the emergence of tumors. Notably, signaling is a key role of Tregs in wound healing, as seen through the EGFR axis; however, this process can be hijacked to promote tumorigenesis (156).

### 7.2 Treg-targeting strategies in cutaneous wounds

Based on our understanding of the immune system’s role in skin tissue regeneration, targeting the immune system offers a promising approach for developing innovative regenerative strategies. Various immune-mediated therapeutic patterns in treating cutaneous wounds have been studied and summarized elsewhere. These major strategies include the use of cytokines, protease inhibitors, miRNA, small interfering RNA (siRNA), and extracellular vesicles (EVs) aimed at promoting re-epithelialization and angiogenesis, recruiting progenitor cells, directing macrophage polarization, and inhibiting excess inflammation (25). Modulating the stability and functions of Tregs, as part of the adaptive immune system, can lead to the previously listed functions.

Potential Treg-based therapies to increase Treg recruitment or activity in wounds are being explored to enhance Treg activity in these areas. Strategies to expand or activate endogenous Tregs at the wound site could restore balance to the inflammatory environment (**Table 2**). This can also promote the sought-after transition from chronic inflammation to effective healing. These strategies may include topical application of cytokines and growth factors. In skin tissue regeneration, endogenous Tregs can be targeted by topically introducing cytokines and growth factors that can induce Treg stability and functions. Growth factors and cytokines, including EGF, PDGF, IL-10, and Areg, can promote tissue repair by modulating the degree of inflammation (165, 166). As already stated, Areg and EGF can stimulate the EGFR pathway, which is involved in the regeneration of the epidermis and dermis. In addition to its antioxidant and catalytic functions, the novel metal-polyphenolic nanozyme (Zn-DHM NPs) reprogrammed the Th17/Treg ratio by upregulating Foxp3 and consequently attenuated the IL-17 signaling pathway in diabetic wounds. This shift led to the differentiation of naive CD4+ T cells into Tregs (161).

**Table 2:** Targeting skin Tregs to promote wound healing.

Strategies	Examples	Mechanisms	Effects	Reference
Adoptive Treg cell therapy	Recombinant Tregs CAR-Tregs	Tregs adopt injury-specific phenotypes.	Delivery of exogenous Tregs to increase frequency and reduce inflammation	(21)
Cytokine and growth factor-based approaches	Low-dose IL-2/anti-IL-2 complexes IL-10 therapy Sustained IL-33 release EGF, Areg, PDGF, VEGF, FGF	M2 macrophage polarization Amplification of ST2-expressing Modulation of EGFR and TGFβ signaling pathways	Topical application of cytokines and growth factors to enhance the activation, function, amplification, and survival of endogenous Tregs enhance ROS clearance	(159)
Pharmacological agents (metabolic regulators and natural compounds)	Rapamycin Curcumin	Epigenetic modifiers such as Vitamin D and histone deacetylase inhibitors Curcumin produces TGF-β1	Local administration of these agents promotes the proliferation of Tregs and simultaneously inhibits effector T cells Natural molecules could influence Treg cell activities	(160)
Physical factors	UVB irradiation	Secretion of proenkephalin and amphiregulin	Expansion of Tregs with skin repair functions	(126)
Endogenous Treg cell recruitment	Cell-based treatment Biomaterial scaffolds Nanozymes	Mesenchymal stem cells secrete factors (e.g. IL-10, TGF-β1) Chitosan-based delivery of neurotensin Sulfated polysaccharide Zn-dihydromyricetin (Zn-DHM) nanoparticle	inflammation resolution by downregulating Th17 and increasing Treg differentiation promote angiogenesis facilitation of macrophage-Treg crosstalk	(161–164)

Another strategy is the introduction of exogenous Tregs. A growing body of research suggests that, in addition to local endogenous Tregs, which include some that are dysfunctional, Tregs can be introduced into the wound bed to modulate inflammation and promote wound healing. As a type of immunotherapy, adoptive Treg transfer has shown limited success due to the low frequency of Tregs in the peripheral blood, as discussed earlier (167). The use of autologous cells from patients with chronic wounds, whose Tregs may be dysfunctional, the lengthy *in vitro* expansion of Tregs, cell delivery and survival in the inflammatory wound environment, and the absence of standardized generation protocols are all drawbacks of adoptive Treg therapies (168). As a result, neither FDA-approved treatments nor registered clinical studies exist for using adoptive Tregs therapy to manage chronic, non-healing wounds. The functional use of Treg-based treatments has only been investigated in pre-clinical and *in vitro* settings. The bench-to-bed application must still be thoroughly studied (169). These are challenges that can be overcome in subsequent research.

In some literature, pharmacological agents, in addition to growth factors, small-molecule activators, or inhibitors, can be introduced. These agents can manipulate the molecular characteristics and Tregs signaling pathways. Multiple small natural compounds exhibit anti-inflammatory, anti-diabetic, and antimicrobial properties, and can promote wound healing (170). Recently, the mechanistic activities of small molecules that increase ‘Tregness’, such as Oleracein E, Indole-3-aldehyde, and Eupalitin-3-O-β-D-galactopyranoside, were studied. Some of these Treg proliferators seem to act through a site different from the canonical mTOR pathway, as well as the rapamycin and STAT5 pathways (171). These compounds can modulate Treg stability and functions through various signaling pathways, especially in chronic wounds. In comparison to other immune cells, studies on therapeutically targeting T cells, particularly Tregs, are limited. Due to their low cost and easy accessibility, studies on the effects of natural compounds on wound healing can be encouraged.

While these strategies hold promise in wound therapy, their execution may be challenging due to the complex wound environment. Together, hypoxia, glycolysis, and lactylation establish a dysfunctional metabolic program and cellular communication in DFU, and that can alter potential treatments (154). A foundational study established that in hypoxic conditions, HIF-1α could reprogram expanded Tregs to a pro-inflammatory phenotype (102). Therefore, to mitigate this risk in therapeutic strategies, the design of delivery systems should prioritize materials capable of not only expanding Tregs and boosting their functions, but can arming them against plasticity. For instance, some nanoparticles also have intrinsic anti-inflammatory and glucose-diminishing properties (161), brain-derived neurotrophic factor (BDNF) hydrogel preserved Foxp3 and also regulated macrophage polarization in nerve regeneration (172), hyaluronic acid and methyl cellulose (HAMC) Tregs had twofold increased chances of penetrating the ocular inflammatory environment in experimental autoimmune uveitis (EAU) (173).

## 8 Summary and future perspectives

Effective wound healing is crucial for restoring the skin’s barrier function following tissue injury, thereby preventing infections and chronic conditions. Key factors in successful healing include reducing inflammation, scarring, and fibrosis. Tregs are vital in tissue repair, as they regulate hemostasis, attenuate the accumulation of pro-inflammatory cells and factors, and support the transition from the inflammatory to the proliferative phase of healing by converting pro-inflammatory macrophages into pro-repair, anti-inflammatory macrophages. These macrophages are essential for preventing the progression from acute to chronic wounds. By preferentially expressing specific surface molecules and tissue-specific transcription factors, Tregs can influence the extravasation, motility, stability, and functions of other cells, thereby enhancing cutaneous wound repair. Their interactions with various immune and non-immune cells in the wound microenvironment create a complex network that may be leveraged for advancements in regenerative medicine. Targeting strategies, including the modulation of endogenous Treg signaling pathways, local administration of exogenous Tregs, and topical application of cytokines and growth factors, can enhance their regenerative functions and promote activation and accumulation at the wound site.

There are limitations in ongoing research. The lack of specific markers to identify different immune cell subsets hinders the development of targeted therapies. While single-cell genomics could clarify lineage-specific markers and the roles of various Tregs subsets in tissue repair, imaging studies have shown a significant separation between HFSCs and Tregs in wound beds, raising questions about their interactions (133). Additionally, variations in skin tissue structure, healing ability, and resident microbiota based on body site can significantly influence immune system involvement in wound healing and impact the choice of model organisms for studies (80). Furthermore, the hypothesis that human skin Tregs modulate inflammation through the expression of ARG2, which affects arginine availability for Teff cells, was not supported by findings in mouse skin Tregs, which showed minimal Arg2 expression (84).

Exploring the close contact between skin cells and the peripheral nervous system, numerous studies suggest that neurotransmitters, including neurotrophins and neuropeptides, can influence the immunoregulatory functions of various skin immune cells. For instance, neuritin levels decrease over time with increasing exposure to high glucose. As a neurotrophic factor found to enhance Treg function in certain autoimmune disease settings and promote angiogenesis (174–176), this suggests that Treg plays a key role in chronic wound healing.

Looking forward, while genome editing in Tregs is a subject of much research presently, replicating this technology *in vivo* is necessary. Knoedler et al. pointed out that Tregs outfitted with chimeric antigen receptors (CAR-Tregs) may offer promising therapeutic options for wound healing due to their demonstrated ability to regulate alloimmune-mediated rejection in human skin grafts (22). This may also assist in studies that involve the transfer of exogenous Tregs to treat chronic wounds. Further research is required to determine the optimal timing and delivery strategy for administering these exogenous Tregs, as effective cutaneous repair may not be achieved otherwise. The relationship between Tregs and the pathophysiology of diabetic wounds could serve as a basis for future research.

Additionally, understanding the pathophysiological relationship between Tregs and diabetic wounds could provide valuable insights for future research. Recent findings indicate that specific microRNAs, such as miR-92a, may influence Treg activity, highlighting a potential strategy for enhancing their functions (177). Although some studies have explored the modulation of Tregs in various contexts, including tumors (178) and diverse immune cells in wound healing (179), a notable gap remains in research regarding the impact of microRNAs on Tregs in wound healing. This is a potential strategy for future research. While this approach is utilized, it is also important to note that different populations of Tregs coexist in the skin and appear to serve diverse purposes, as seen in cases where subsets of skin Tregs and neutrophils are involved (180). Finally, the diversity among Treg populations in the skin warrants further investigation. Understanding the distinct roles of these subsets, particularly those involving hair follicle stem cells (HFSCs) during wound healing, could offer new insights into Treg functionality and therapeutic applications (133).
